# Efficacy of Oral Care Protocols in the Prevention of Ventilator-Associated Pneumonia in Mechanically Ventilated Patients

**DOI:** 10.7759/cureus.23750

**Published:** 2022-04-02

**Authors:** Pallika Singh, Zia Arshad, Vinod K Srivastava, Gyan Prakash Singh, Radhey S Gangwar

**Affiliations:** 1 Anesthesiology, Autonomous State Medical College, Firozabad, IND; 2 Anesthesiology and Critical Care, King George's Medical University, Lucknow, IND; 3 Geriatrics Mental Health, King George's Medical University, Lucknow, IND

**Keywords:** oral hygiene, control group, boas score, dental plaque, chlorhexidine, ventilator associated pneumonia

## Abstract

Background: Ventilator-associated pneumonia (VAP) is one of the most common infections in intubated intensive care unit (ICU) patients. Oral care with chlorhexidine is a conventional method for maintaining hygiene. Recently, adjuvant methods have been introduced into routine oral care, including teeth brushing and the application of moisturizing lotion. The objective of this study was to compare the incidence of VAP in critical care patients receiving oral care with and without manual teeth brushing and the application of moisturizers to the mouth.

Methods: We conducted a prospective randomized control study comprised of 220 ICU patients between 18 and 65 years of age, and of either sex. The patients were divided into two groups of 110 each. Care for the study group (group S) consisted of chlorhexidine wash, tooth brushing, and moisturizing gel over gums, buccal mucosa, and lips. The control group (group C) was treated with chlorhexidine wash only. The oral assessment was done at 4, 6, 8, and 12 hours using the Beck Oral Assessment Scale (BOAS). Pneumonia was assessed based on abnormal chest x-rays, fever, chest auscultation, endotracheal culture report, and the incidence of VAP, and mortality was observed

Results: Abnormal chest x-rays, positive auscultatory findings, fevers, and positive culture reports were significantly reduced in group S compared to these measurements in group C. The incidences of VAP and mortality were also significantly lower in group S compared with the incidences in group C.

Conclusions: Oral care with chlorhexidine mouth wash and the adjuvant measures reduced VAP and, consequently mortality and hospital stays. Tooth brushing along with standard oral care provides an additional advantage in the prevention of VAP in mechanically ventilated patients. Compulsory tooth brushing, if included in regular oral care yields better results in terms of decreased incidence of VAP, length of ICU stay, and mortality.

## Introduction

Ventilator-associated pneumonia (VAP) is defined as pneumonia that develops at least 48 h after endotracheal intubation and initiation of mechanical ventilation [1]. VAP is the second most common nosocomial infection after urinary tract infection and the most common infection in mechanically ventilated intensive care unit (ICU) patients [2]. VAP is a major medical problem with a mortality rate between 33% and 50% [3].

The endotracheal tube (ETT) acts as a pathway for spreading pathogenic bacteria from the oral cavity. Microaspiration of pharyngeal secretions may occur due to improper sealing of the ETT cuff in ventilated patients. Microaspiration can cause nosocomial pneumonia [4]. Poor oral health is a common problem in mechanically ventilated patients [5]. Dental plaque is an archetypal biofilm, which is rapidly colonized by potential respiratory pathogens in critically ill patients; thus, dental plaque is a reservoir for VAP pathogens [6-9]. Dental plaque is increased and forms faster in ICU patients compared with other patients [10]. The oral flora changes in the first 48 h of hospitalization and is replaced mainly by Gram-negative organisms. The growth of these organisms leads to dental plaque formation [11]. The accumulation of aerobic and anaerobic microorganisms contributes to the growth of plaque mass. Colonization by Gram-negative bacteria is crucial to the accumulation of oral and pharyngeal bacteria [12,13]. The bacteria in dental plaque cause ventilator-associated pneumonia (VAP) [10,14].

Randomized clinical trials demonstrated that improving oral hygiene reduces VAP and mortality [15-17]. However, these trials did not measure oral cleanliness. Furthermore, a paucity of research conducted in mechanically ventilated patients has focused on the optimal methods for improving oral hygiene [18]. According to Eilers’s Oral Assessment Guide, numerous commercially available oral care products, such as manual and electric toothbrushes, dentifrices, oral moisturizing agents, and a variety of oral swabs and solutions, are available [19]. The effectiveness of most of these products and methods on oral health is not well known, especially in the context of intubated patients. Routine oral hygiene is encouraged in mechanically ventilated patients to reduce VAP incidence, but evidence supporting specific strategies is limited.

The aim of this study was to correlate oral hygiene with or without adjuvant oral care with VAP in ICU patients. The incidence of VAP was compared between a study group receiving adjuvant oral care along with routine oral hygiene and a control group receiving only oral hygiene.

## Materials and methods

The Institutional Ethics Committee of King George's Medical University (KGMU) approved this study (reg. no.: ECR/262/Inst/UP/2013/RR-19). This randomized control study was conducted in ICU patients at King George's Medical University over one year (May 2019 to April 2020). Incidence of VAP, mortality, and duration of hospital stay were compared between groups. The inclusion criteria were ICU patients aged 18-65 years of either sex with oral ETT in situ. The exclusion criteria were more than 48 hours of mechanical ventilation before ICU admission, previous history of respiratory illness, immunocompromised, ongoing sepsis, pregnancy, and lack of denture. After strictly following the inclusion and exclusion criteria, 220 ICU patients were enrolled in the study. Sample size was calculated on the basis of the rate of ventilator-associated pneumonia before and after the intervention using the following formula.

n = D ({Zα + Zβ}^2^ / {In(1-e)}^2^ × {P1 (1 - P1) + P2 (1 - P2)} / {P2 - P1}^2^)

**Figure 1:**
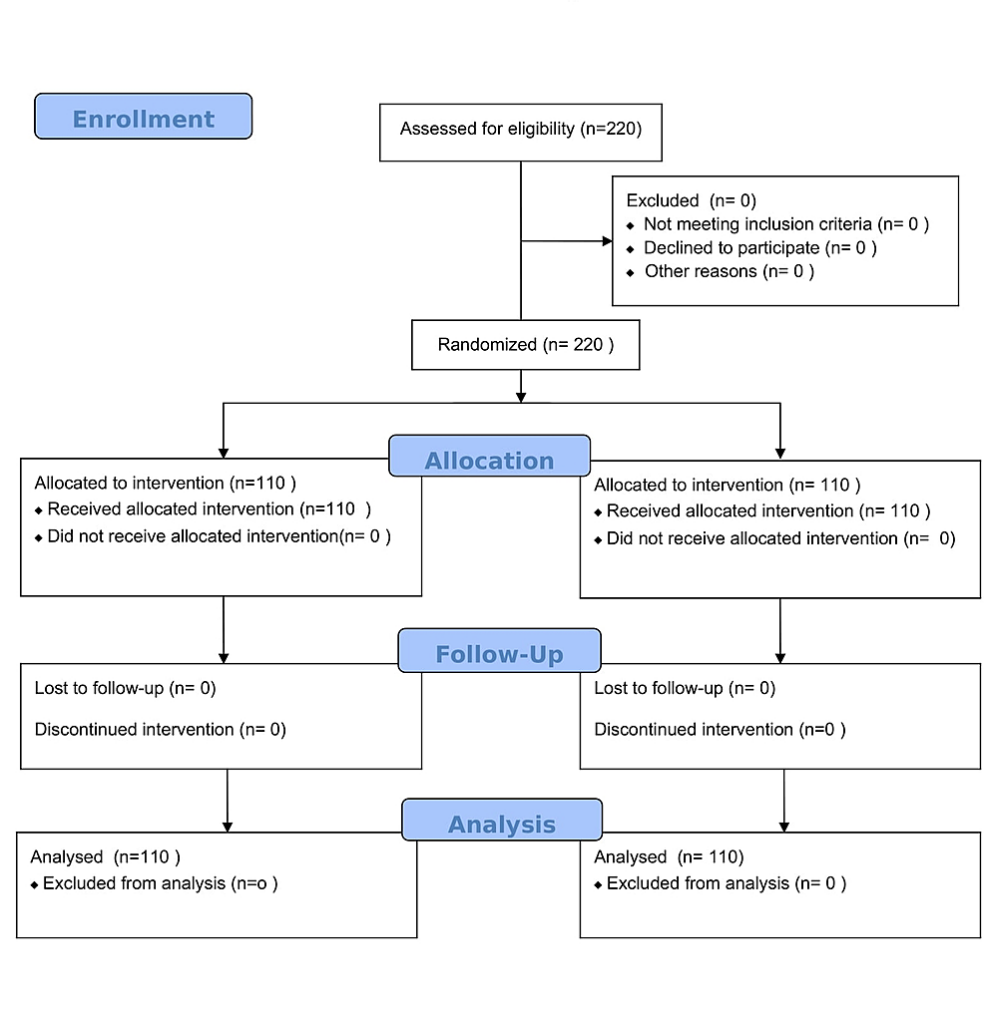
Consort diagram showing randomization, allocation, follow-up, and analysis of enrolled patients.

Here, p_1_=0.012 is the rate of ventilator-associated pneumonia before the intervention, p_2_=0.008 is the rate of ventilator-associated pneumonia after the intervention [20]. Coefficients difference e=0.9 is considered to be clinically significant, type 1 error (α=5%), type 2 error (β=20%) for setting power of study 80%, the minimal sample size is calculated to be n=215. Randomization and follow-up of the cases in the study are shown in the consort diagram (Figure 1).

Randomization was done using a computer-generated random number. First time, the Beck Oral Assessment Scale (BOAS) score was taken at four hours, so this can be considered as baseline. The following interventions were performed twice a day in group S: the ETT cuff pressure was maintained between 20 and 25 mmHg by using Posey Portex ET tube cuff manometer (Karnataka, India: Alpha Biomedix); the head end of the bed was elevated 30°-45°; deep mouth and throat suctioning were performed; oral hygiene was maintained by brushing the outer and inner surfaces of teeth, gums, and tongue using a baby brush with antimicrobial chlorhexidine 0.2%; moisturizing gel containing aloe vera and peppermint oil was applied over the oral mucosa, gums, and tongue and petroleum jelly was applied to the lips; and the airway was examined, any obstructions were removed, and if necessary, the tube was changed. In group C, all routine oral care was performed as in the study group except manual tooth brushing; application of moisturizing gel over the oral mucosa, gums, and tongue; and application of petroleum jelly to the lips (Table 1).

**Table 1:** Intervention in study and control group. VAP: ventilator-associated pneumonia; ET: endotracheal tube; DVT: deep vein thrombosis

S.N.	Group S	Group C
1.	VAP bundle: raised the head end of the bed 30°-45°, daily sedation hold, DVT prophylaxis, peptic ulcer prophylaxis	VAP bundle: raised head of bed 30°-45°, daily sedation hold, DVT prophylaxis peptic ulcer prophylaxis
2.	ET tube cuff pressure 20-25 mmHg	ET tube cuff pressure 20-25 mmHg
3.	Chlorhexidine mouthwash 0.2 %	Chlorhexidine mouthwash 0.2 %
4.	Tooth brushing or gauze piece	Nil
5.	Lubrication of oral mucosa	Nil

The observation was started from the first day of admission to the ICU. Oral care was assessed using the Beck Oral Assessment Scale(BOAS) score and categorized into four types [21]. If the BOAS score was 0-5, oral assessments were recommended once a day but oral care was performed twice a day as mentioned in the systematic oral care procedure. If the BOAS score was 6-10, oral assessments were recommended twice a day, and mouth/lips were moistened every four hours. In this condition, oral care was also performed twice a day as outlined in the systematic oral care procedure. If the BOAS score was 11-15, oral assessments were recommended during every eight hours shift. Oral care was performed as outlined in the systematic oral care every shift. In all patients of the study group, an ultra-soft toothbrush was used and the lips and mouth were moistened every two hours. If the BOAS score was 16-20, oral assessments were recommended every four hours. If brushing was not possible, a soft gauze wrapped around the fingers was used to moisten the lips and mouth every one to two hours (Table 2).

**Table 2:** Beck Oral Assessment Scale (BOAS) (modified). Provide moisture more than oral care. The table is obtained with permission from Gupta et al. [22].

Area	Score			
	1	2	3	4
Lips	Smooth, pink, moist, and intact	Slightly dry, red	Dry, swollen isolated blisters	Edematous, inflamed blisters
Gingival and oral mucosa	Smooth, pink, moist, and intact	Pale, dry, isolated lesions	Swollen red	Very dry and edematous
Tongue	Smooth, pink, moist, and intact	Dry, prominent papillae	Dry, swollen, tip and papillae are red with lesions	Very dry, edematous, engorged coating
Teeth	Clean, no debris	Minimal debris	Moderate debris	Covered with debris
Saliva	Thin, watery plentiful	Increase in amount	Scanty and somewhat thicker	Thick and ropy, viscid or mucid
Total score	5, no dysfunction minimum care every 12 hrs	6-10, mild dysfunction minimum care every 8-12 hrs	11-15, moderate dysfunction minimum care every 8 hrs	16-20, sever dysfunction minimum care every 4 hrs

Statistical analyses were performed using Statistical Package for Social Sciences (SPSS) version 21.0 statistical analysis software (Armonk, NY: IBM Corp.). The values are represented as numbers (%) and mean ± SD. The sample size was calculated based on an α risk of 0.05 and β risk of 0.20 (80% power of the study). Comparisons between groups at different time intervals were assessed using Student’s t-test. All categorical data were compared using chi-square tests. A p ≤ 0.05 was considered statistically significant.

## Results

Demographic profiles were comparable in both groups (p=0.796). All patients received 0.2% chlorhexidine mouthwash at regular intervals, starting from admission into the ICU and repeated every day for up to five consecutive days. Of the 220 patients enrolled in the study, 110 (50.0%) patients were in group S and 110 patients were in group C. The demographic profiles were similar between the groups (p = 0.796) (Table 3).

**Table 3:** Comparison of demographic profiles between the groups in study population NS: not significant

	Group S (n=110)		Group C (n=110)		Total (N=220)	
Mean age in years ±SD (Range)	39.02±16.87 (18-65)		39.10±14.21 (18-65)		39.06±15.56 (18-65)	
t=0.048; p=0.969 (NS)						
Gender	No.	%	No.	%	No.	%
Female	55	50.0	54	49.1	109	49.5
Male	55	50.0	56	50.9	111	50.5
χ2=3.856 (df=1); p=0.796						

The majority of the overall patients (62.3%) as well as of group S (64.5%) and group C (60.0%) did not suffer from any comorbidity. Though comorbidities were present in higher proportion of patients of group C (40.0%) as compared to group S (35.5%), this difference was not found to be significant statistically (Table 4). Results show the patients with comorbidity in both groups are comparable (p=0.487) (Table 5). Fever was found in a significantly higher proportion of group C cases as compared to group S (75.5% vs. 42.7% and 81.8% vs. 6.8%) (Tables 6). Range of oxygen saturation level in the overall study population was 68-100%. In group S, level of oxygen saturation was higher than group C (95.75±6.09% vs. 92.88±7.94%) which was statistically significant (Table 7).

**Table 4:** Comparison of prevalence of different comorbidities between the groups in study population OSA: obstructive sleep apnea

S.N.	Comorbidities	Total (N=220)	Group S (n=110)		Group C (n=110)		Significance of differences	
			No.	%	No.	%	χ2	p-value
1	Diabetes	46	20	18.2	26	23.6	0.990	0.320
2	Hypertension	57	28	25.5	29	26.4	0.024	0.878
3	Hypothyroidism	6	3	2.7	3	2.7	0.000	1.000
4	OSA	1	0	0.0	1	0.9	1.005	0.316

**Table 5:** Comparison of comorbidities between the groups in study population

S.N.	Comorbidities	Group S (n=110)		Group C (n=110)		Total (N=220)	
		No.	%	No.	%	No.	%
1.	No comorbidities	71	64.5	66	60	137	62.3
2.	Comorbidities	39	35.5	44	40	83	37.7
χ2=0.484 (df=1); p=0.487							

**Table 6:** Comparison of incidence of fever between the groups in study population

S.N.	Risk factors	Total (N=220)	Group S (n=110)		Group C (n=110)		Significance of differences	
			No.	%	No.	%	χ2	p-value
1	Fever	130	47	42.7	83	75.5	24.369	<0.001

**Table 7:** Comparison of oxygen saturation level between the groups in study population

Group	No. of cases	Min	Max	Mean	SD
Group S	110	70	100	95.75	6.09
Group C	110	68	100	92.88	7.94
Total	220	68	100	94.31	7.20
t=3.001 (Student t-test); p=0.003					

The BOAS score was assessed at four, six, eight, and 12 hours after beginning the oral hygiene routine. The BOAS scores of patients in group S were significantly lower than the patients in group C for all-time points. In group S, a significant decline in BOAS scores (at four hours) was observed from baseline to six, eight, and 12 hours after beginning the oral hygiene routine (Table 8).

**Table 8:** Comparison of Beck Oral Assessment Scale scores between the groups in study population

S.N.	Time interval	Group S (n=110)		Group C (n=110)		Student t-test	
		Mean	SD	Mean	SD	t-value	p-value
1	At 4 hours	11.55	3.32	13.11	2.66	-3.837	<0.001
2	At 6 hours	10.74	3.18	13.21	2.85	-6.073	<0.001
3	At 8 hours	9.56	2.63	13.49	2.89	-10.528	<0.001
4	At 12 hours	9.09	2.59	13.58	2.80	-12.341	<0.001

Normal chest x-ray was observed for significantly higher proportion of cases of group S as compared to group C (47.3% vs. 23.6%) (Table 9). Endotracheal tube culture of group S (66.4%) was found to be sterile while in group C (59.1%) was found to be infected which was significant statistically (Table 9).

**Table 9:** Comparison of investigation findings between the groups in study population CXR: chest x-ray; ETT: endotracheal tube

S.N.	Investigation	Group S (N=110)		Group C (N=110)		Total (N=220)	
		No.	%	No.	%	No.	%
1.	Normal CXR	52	47.3%	26	23.6%	78	35.5%
2.	Abnormal CXR	58	52.7%	84	76.4%	142	64.5%
χ2=13.427 (df=1); p<0.001							
3.	ETT culture (sterile)	73	66.4	45	40.9	118	53.6%
4.	ETT culture (infected)	37	33.6	65	59.1	102	46.4%
χ2=13.427 (df=1); p<0.001							

Fever, ETT pathogens, and chest x-ray abnormalities are features of VAP. The presence of these features indicates VAP. The prevalence of VAP was significantly higher in group C compared with the prevalence of VAP in group S (47.3% vs. 29.1%). Overall, 220 patients completed the study. The mortality rate was significantly higher in group C (60.0%) compared with the mortality rate in group S (44.5%). The mortality was due to VAP and its subsequent complication. The remaining cases were transferred from ICU to the wards or discharged (Table 10).

**Table 10:** Comparison of final outcome between the groups in study population VAP: ventilator-associated pneumonia

S.N.	Final outcome	Group S (n=110)		Group C (n=110)		Total (N=220)	
		No.	%	No.	%	No.	%
1	VAP	32	29.1	52	47.3	84	38.2
2	No VAP	78	70.9	58	52.7	136	61.8
χ2=7.703 (df=1); p=0.006							
1	Discharged from ICU	61	55.5	44	40.0	105	47.7
2	Expired	49	44.5	66	60.0	115	52.3
χ2=5.265(df=1); p=0.022							

Both ICU stays and mechanical ventilation requirements were significantly higher in group C compared with ICU stays and mechanical ventilation requirements in group S (ICU stays: 15.05 ± 9.49 vs. 11.85 ± 7.44 days; mechanical ventilation: 14.35 ± 9.47 vs. 10.94 ± 7.59 days) (Table 11).

**Table 11:** Length of ICU stay and number of days of mechanical ventilation

	Group S (n=110)		Group C (n=110)		t-Value	p-Value
	Mean	SD	Mean	SD		
ICU stay (days)	11.85	7.44	15.05	9.49	-2.78	0.006
Days of mechanical ventilation	10.94	7.59	14.35	9.47	-2.95	0.003

## Discussion

In our study, the age of patients ranged from 18 to 65 years, and the mean age of patients was 39 years. The male-to-female ratio was 1:1 in both intervention groups. No differences in age or sex were detected between the two groups. In the study by Lorente et al., the mean patient ages were 61 and 60 years, respectively, in the tooth brushing and non-tooth brushing groups, and the male-to-female ratio was 3:1 in both groups [23]. In the Lev et al. study, the mean age was 71.8 years (SD=14.8) in both groups and the male-to-female ratio was 1.2:1 and 1.1:1 in the study and control groups, respectively [24].

In our study, 37.7% of patients had comorbidities and the proportion of patients with comorbidities was higher in group C (40.0%) compared with the proportion in group S (35.5%), but this difference was not statistically significant (p=0.487). In the study of Atashi et al., comorbidities were present in 53.9% of patients and were higher in the placebo group (60.50%) than in the intervention group (47.40%) [25].

Our study assessed the BOAS score at four, six, eight, and 12 hours after beginning oral hygiene. At all-time points following the initiation of oral care, the BOAS scores were significantly lower in group S than the BOAS scores in group C. In group C, a subsequent increment in baseline BOAS scores (at four hours) was observed at six, eight, and 12 hours. Minimal changes in BOAS scores of 0.10 ± 1.37 were observed at six hours (0.76%) that reached 0.47 ± 1.25 at 12 hours (3.61%).

According to the CDC guidelines, we included patients in whom VAP was suspected based on the new onset of abnormal chest x-rays, fever, chest auscultation, positive ETT cultures, or increased ventilator demand. We suspected VAP in 29% of patients in group S and 47.3% of patients in group C. The prevalence of VAP was significantly higher in group C compared with the prevalence in group S. Atashi et al. showed that the incidence of pneumonia in the study group was lower but not statistically different from the incidence in the control group [25]. Various studies, including Fourrier et al. in 2000, Houston et al. in 2002, Grap et al. in 2004, and Koeman et al. in 2006, support our results [26-29]. These studies all demonstrated that the rate of VAP was reduced in the intervention group compared with the rate in the control group. However, Lorente et al. found no significant difference in the two groups [23].

In our study, the ETT cultures in 33.6% of patients in group S were infected compared with 59.1% infected ETT cultures in group C. Lorente et al. showed that 9.6 % of ETT cultures were infected in the tooth brushing group, whereas 10.95% of ETT cultures were infected in the non-tooth brushing group [23].

In our study, the mean ICU stay was 11.85 ± 7.44 days and 15.05 ± 9.49 days in groups S and C, respectively. The duration of mechanical ventilation was 10.94 ± 7.59 and 14.35 ± 9.45 days in groups S and C, respectively. Lorente et al. demonstrated similar findings; the intervention decreased the mean duration of ICU stays and mechanical ventilation [23]. In our study, mortality was significantly higher in group C (non-tooth brushing group) (60%) compared with mortality in group S (tooth brushing group) (44.5%). While in the study of Lorente et al., the mortality rates were 28.6% and 31.5% in the tooth brushing and non-tooth brushing groups, respectively [23]. Munro et al. showed that the mortality rate was 20% in the tooth brushing only group, 30% in the chlorhexidine only group, 25% in the tooth brushing and chlorhexidine group, and 18% in the control group [30]. This difference in mortality rates may be due to the poor condition of patients at our hospital; our hospital is a tertiary care hospital in which most patients are referred in poor condition with grave prognoses.

The present study has some limitations. Our study was performed in a single ICU. Therefore, the results may vary at different ICUs, and the results cannot be applied uniformly. For those patients who were admitted from outside, the history of previous antibiotic use and the guidelines for intubation were unavailable. A further limitation of the study is that the VAP diagnostic procedure was not invasive; we used only tracheal aspirate samples.

## Conclusions

VAP is a major clinical problem for critically ill patients. Further research to prevent VAP is needed. Based on the results of our study, we conclude that tooth brushing along with oral care provides an additional advantage in preventing VAP in patients on mechanical ventilation. Different studies on tooth brushing might yield different results, and more studies are needed to formulate evidence-based guidelines for oral care to minimize the incidence of VAP. Compulsory tooth brushing decreased not only the number of VAP patients but the length of ICU stays and the ventilator time. Finally, mortality was lower in patients who received tooth brushing along with oral care.
